# Testing a vaccine candidate against Hepatitis C virus designed by combinatorial optimization

**DOI:** 10.1038/s41598-023-48458-x

**Published:** 2023-12-08

**Authors:** Iker Malaina, Luis Martinez, David Salcines-Cuevas, Hector Teran-Navarro, J. Gonzalo Ocejo-Vinyals, Elena Gonzalez-Lopez, Vicente Soriano, María Ubeda, Martin-Blas Perez Pinilla, Ildefonso Martinez de la Fuente, Carmen Alvarez-Dominguez

**Affiliations:** 1 Department of Mathematics, University of the Basque Country (UPV/EHU), 48080 Bilbao, Spain; 2 Biocruces Health Research Institute, Bilbao, Spain; 3 Basque Center of Applied Mathematics (BCAM), 48009 Bilbao, Spain; 4 Facultad de Ciencias de La Salud, Universidad Internacional de La Rioja (UNIR), MEDONLINE Group, Avda. de La Paz, 137, 26006 Logroño, La Rioja Spain; 5 Servicio de Inmunología, Cantabria and Instituto de Investigación Marqués de Valdecilla (IDIVAL), Hospital Universitario Marqués de Valdecilla, Avda. de Valdecilla S/N, 39008 Santander, Spain; 6 Centro de Edafología y Biología Aplicada del Segura, 30100 Murcia, Spain

**Keywords:** Protein design, Hepatitis C, Applied mathematics

## Abstract

This paper presents a new procedure for vaccine design against highly variable viruses such as Hepatitis C. The procedure uses an optimization algorithm to design vaccines that maximize the coverage of epitopes across different virus variants. Weighted epitopes based on the success ratio of immunological assays are used to prioritize the selection of epitopes for vaccine design. The procedure was successfully applied to design DC vaccines loaded with two HCV peptides, STG and DYP, which were shown to be safe, immunogenic, and able to induce significant levels of anti-viral cytokines, peptide-specific cellular immune responses and IgG antibodies. The procedure could potentially be applied to other highly variable viruses that currently lack effective vaccines.

## Introduction

With 58 million people infected with chronic hepatitis C virus (HCV) and approximately 290,000 people dying in 2019^1^, hepatitis C is one of the main global health burdens as it is responsible not only for viral infections, but also for other chronic hepatitis and liver diseases with a considerable health impact, such as cirrhosis, hepatocellular carcinoma and liver transplantation^2^. According to the last the Annual Epidemiological report of the European Center for Disease Prevention and Control (ECDC) in 2020^3^, 13,914 cases were reported in the 28 EU member states, implying a rate of 3.9 cases per 100,000 people. Only 6% of the cases corresponded to acute HCV infections and they are usually asymptomatic or with mild symptoms, including fever, fatigue, icteric and high transaminase levels^2^. Approximately 35% are classified as severe chronic cases, primarily associated with cirrhosis and hepatocarcinoma. However, a significant proportion, around 53%, of HCV cases fall under the category of "unknown". HCV infection mainly affects the group of young males aged 35–44 and among females aged 25–44^2^. The most commonly mode of transmission was injection drug use, (58% acute and 75% chronic HCV cases), followed by sex between men (25% acute and 5–6% chronic cases), heterosexual transmission (6% acute, 3% chronic cases), nosocomial (5% acute, 8% chronic), non-occupational injuries such as needle-sticks, bites, tattoos and piercings (4% acute, 6% chronic cases), and blood and blood products (2% acute, 6% chronic cases). Moreover, variations in the surveillance systems hinder the definition of reported cases as acute or chronic, but also regarding the criteria to differentiate between acute and chronic hepatitis C^2^. In this regard, the latest epidemiological surveillance in Spain in 2020 showed a hepatitis C incidence of 2.36 cases per 100,000 inhabitants^4^ with 7.8% being new acute HCV cases and 56.3% new chronic cases. Moreover, 2.9% of the chronic cases presented coinfections with other viruses such as hepatitis B and hepatitis A viruses and HIV. The recent direct-acting antivirals (DAA) have allowed the cure of most HCV carriers and diminished the interest in developing a vaccine for HCV. However, the number of cases with HCV re-infection, the difficulties for a surveillance system common in all European countries and the different at-risk populations have set back the efforts to search for HCV vaccines^5^. Accordingly, the World Health Organization is committed to eliminating or reducing new HCV infections by 90% by 2030, and an HCV vaccine candidate will aim to accelerate this achievement by preventing transmissions as well as reducing HCV-associated diseases^6^ .

HCV belongs to the Flaviviridae family and has a size of 60 nm. It has a single chain RNA of positive polarity and an icosahedral nucleocapsid composed of C protein and an envelope with two glycoproteins, E1 and E2; these three proteins are considered structural proteins. The RNA also codifies for several non-structural proteins, two transmembrane proteins, NS1, NS2 and metalloprotease NS3, two cofactors NS4A and NS4B, a protein for IFN resistance, NS5A and an RNA polymerase called NS5B. Since NS5B lacks exonuclease 3’-5’ activity to correct errors, HCV shows a high mutational rate. Diagnosis and seroprevalence analysis of HCV is performed with anti-HCV tests in sera that detect antibodies against recombinant antigens of the C nucleocapsid protein, NS3, NS4 or NS5 proteins using an enzyme-immunoassay as biomarkers of chronic infection, while detection of viral RNA by molecular procedures indicates an active infection^7^.

Several issues have impeded the task of preparing a prophylactic vaccine to control HCV^8^. First, HCV is the most variable hepatitis virus with at least 8 different genotypes and more than 90 sub-genotypes^9^. This genetic diversity of HCV is further increased by the error-prone polymerase that generates viral variants within each infected individual that might promote antibody resistance^10^. Second, the requirement of T cell responses for protection – together with B cell responses –is not yet fully understood for HCV infections. Only indirect evidence indicates the existence of protective immunity, such as the spontaneous clearance of HCV infections in 25% of acutely infected individuals or reinfections that are cleared more often than primary infections^11^. In fact, this indirect evidence of immunity supports the hypothesis that adaptive immunity in reinfections is associated with a broader cellular response and the presence of cross-reactive neutralizing antibodies, which highlights the necessity of eliciting both CD4 + and CD8 + T cells to achieve protective immunity for HCV. Third, different limitations to establish HCV culture systems, such as the lack of HCV replication at high levels in nonhuman primate cell lines, have reduced the possibilities of preparing live attenuated and inactivated whole virus vaccines. Fourth, there is limited knowledge on the protein structure of the two envelope glycoproteins E1 and E2, which are highly variable. Furthermore, these envelope proteins, HCV virulence factors, are unknown. Fifth, there is lack of immunocompetent models of small animals to determine whether vaccination induces protective immunity. Finally, an additional difficulty is the selection of those people at high risk to test the vaccine effectiveness, such as people requiring transfusions, injection drug users, and health care workers with frequent exposure to blood or bodily fluids. None of those groups would be feasible to include in cohorts for trials except for the group of health care workers. In fact, the only HCV vaccine clinical trial phase II has so far been conducted with PWID (people who inject drugs) cohort, but despite the promising preclinical results, the vaccine ultimately proved ineffective in protecting against chronic infection, leading to serious concerns regarding our understanding of protective immunity^12^.

Trials of vaccines for HCV have focused on inducing a strong antibody response to protein-based, DNA-based, virus-like particles (VLP), pox virus-based, or whole virus-based vaccines. Most of those vaccine designs involved E1, E2, or NS3 proteins of HCV but have failed to induce sterilizing immunity^8^ or provide high titers of neutralizing antibodies. Several groups have outlined the importance of E2 protein out of all those HCV proteins in the development of vaccines against HCV^5,8,13–16^. Nevertheless, some vaccines – such as VLP-based with E1 and E2 proteins and HCV core – that did not induce antibody responses developed robust T-cell responses^17^. That highlighted the importance of T cell immunity for HCV protection. Therefore, vaccine efforts should focus on inducing cross-reactive antibodies, neutralizing antibodies, and promoting CD4 + and CD8 + T cells to HCV envelope proteins^18^. Prevalence studies of acute and chronic hepatitis C have been performed by detecting RNA by PCR analysis for active HCV infection and anti-HCV antibodies against the nucleocapsid C protein or NS3, NS4 or NS5 proteins. However, those prevalence studies do not detect the glycoproteins E1 or E2 proteins and therefore, they are not adequate methods to follow vaccine effectiveness that measures neutralizing antibodies and antibodies against B cell epitopes. In this vein, computational strategies might aim to propose vaccine designs that combine T and B-cell epitopes and elicit both humoral and cellular immune responses to HCV antigens.

The use of λ-superstrings was introduced by Martinez et al.^19^ as a new computational method to design vaccines and was later generalized^20^ by considering the estimated immunogenicity of each epitope. In short, this method ensures that all the virus variants considered are covered by the vaccine and, consequently, minimizes the capacity of a new variant – derived from any previously considered virus – to infect the patient.

In addition to applying our technique to obtain the vaccine candidate, we only here took experimentally tested epitopes to form our vaccine, instead of considering all possible 9-mers to form the pool of possible MHC class-I epitopes,. Each epitope was weighted according to the degree of success obtained in each assay (T-cell assay, B-cell assay, or MHC assay) performed, with greater relevance being therefore given to those with better chances.

We thus obtained a set of possible vaccine candidates with lengths ranging from 14 to 99 and experimentally tested one of them. When it came to selecting which candidates to evaluate, we bore in mind that some studies indicate that vaccines using long peptides do not appear to induce tolerance and may be more immunogenic than short peptide vaccines^21^. In fact, Sabbatini et al. used peptides with lengths between 30 and 32^22^. Consequently, we analyzed peptides of similar length, up to 40-mer.

The protein chosen to be targeted by our algorithm was the HCV E2 protein, which is the most frequently used target for different vaccines against this virus^14,15,21,23–25^.

After obtaining the set of candidates, we synthesized one of them (subdivided in two peptides) and performed both in vitro and in vivo assays to test its safety, immunogenicity, and effectiveness as vaccine design. Experiments with mice inoculated with dendritic cell (DC) vaccine vectors filled with the peptide candidates and subcutaneously stimulated with the peptides would evaluate local and systemic immune responses. Local immune responses after induction of delayed-type hypersensitivity (DTH) and their characterization seemed to provide information on the immunogenicity of the vaccine designs to elicit significant T cell responses. Meanwhile, systemic immune responses examined in mouse sera (e.g., cytokine patterns and neutralizing antibodies) might confirm their effectiveness as vaccine candidates to elicit broad and cross-reactive immune responses.

In summary, we present and evaluate a new vaccine design procedure based on combinatorial optimization, which offers protection against all variants considered. The epitopes were weighted according to their performance in experimental assays, which brought the optimization process closer to the biological reality. Finally, we experimentally validated two of the obtained peptide candidates as proof-of-concept of efficient vaccines.

## Results

**Figure 1:**
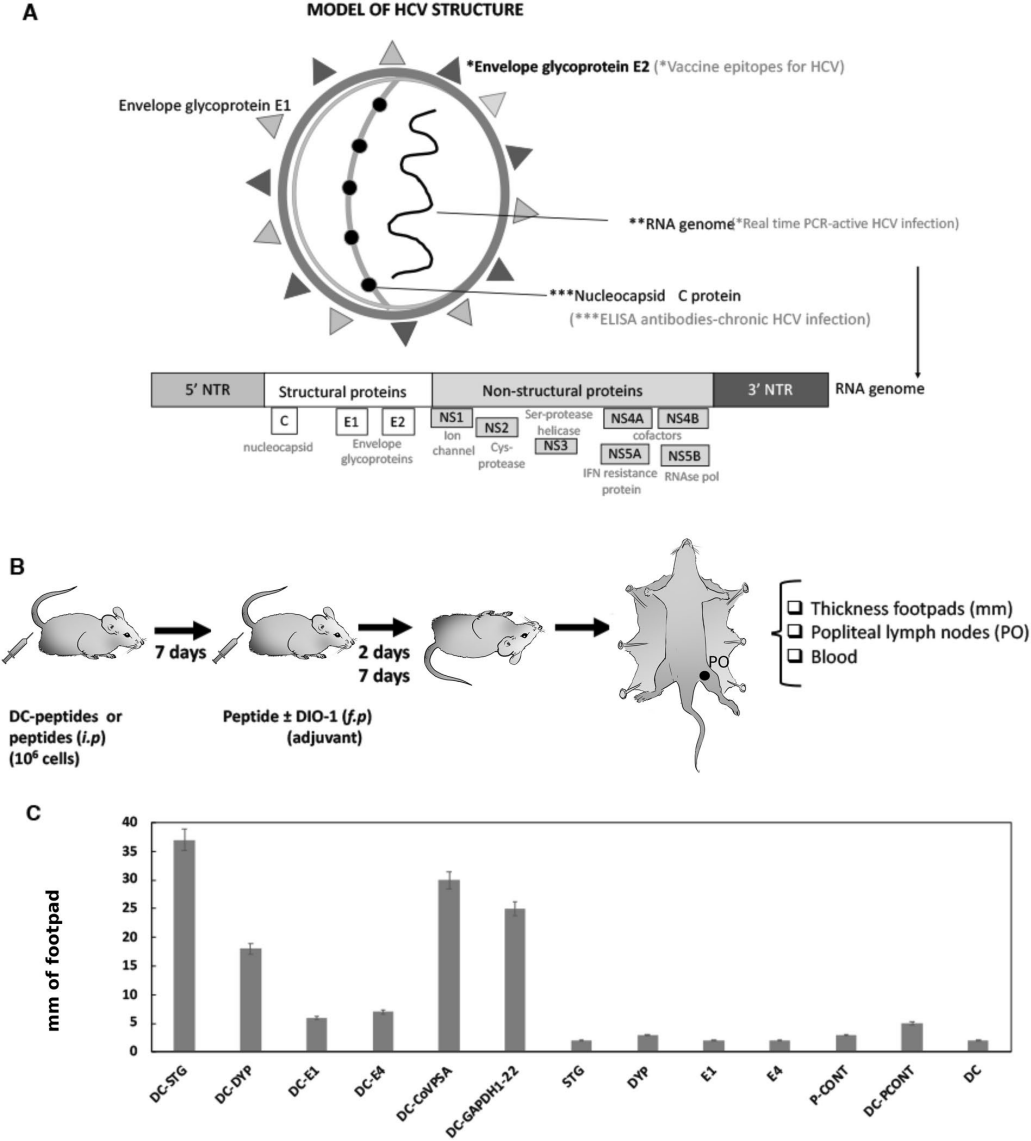
Model of HCV structure for selecting E2 as a vaccine antigen and exploring the immunogenicity of E2 epitopes. (**A**) HCV virus structure and the chosen antigen for vaccine design (indicated by grey * asterisks). (**B**) Demonstration of the immunogenicity of DC vaccines and peptide vaccines in mice: intraperitoneal (i.p.) inoculation of DC or peptide vaccines, followed by peptides injection into the left hind footpads (f.p.), and assessment of footpad swelling. (**C**) Immunogenicity of HCV peptides in vaccine platforms. Hind footpads of mice (C57BL/6, n = 5) were inoculated with DC vaccines (10^6^ cells/mouse) loaded with various peptides (DC-STG, DC-E1, DC-DYP, DC-E4, DC-CoVPSA, DC-GAPDH1-22, or DC-PCONT), as well as empty DC vaccines or peptide vaccines (STG, E1, DYP, E4, CoVPSA, GAPDH1-22, or PCONT) (1 µg/mL per mouse) in formulations with DIO-1 (40 ng/mL). Footpad swelling was measured 48 h later using a caliper and expressed as the difference in millimeters between the left and right hind footpads. The results represent the mean ± SD of three distinct experiments (*P < 0.05).

We first evaluated the structural model of HCV to design vaccine candidates that could include both B and T cell epitopes. Based on the prevalence studies conducted in Spain during the years 2017–2018, anti-HCV antibodies in both acute and chronic hepatitis C cases were only detected against the nucleocapsid C protein in the structure of the virus^26^. However, antibodies produced after an HCV infection would likely recognize epitopes in the envelope proteins of the virus, rather than epitopes of the nucleocapsid. Those epitopes are not available for the antibodies until the virus replicates or phagocytes destroy the viral particles. We chose the E2 protein to target our vaccine candidates to proteins in the envelope, as it is the most frequently used protein in vaccines against HCV (see Fig. 1A for a model).

Once we had selected the HCV antigen – the E2 envelope glycoprotein –, we proceeded to generate vaccine candidates against the Hepatitis C virus. We targeted a set of 1803 virus variants sourced from the European HCV database and considered all the epitopes listed in the Immune Epitope Database and Analysis Resource for the E2 protein of HCV (totaling 439 possible epitopes; see the Methods section for additional details).

Subsequently, we calculated the weights (*w(e)*) associated with each epitope (*e*) using the following approach: Information about T Cell Assays, B Cell Assays, and MHC Ligand Assays is available for every epitope in the IEDB. It should be noted that not all epitopes are associated with all three assay types; thus, certain epitopes might have information only about specific types of assays.

The IEDB website provides a classification of the response obtained into four levels – High-Positive (HP), Intermediate-Positive (IP), Low-Positive (LP), and Negative (N) – for each assay conducted with every epitope. It is important to note that this classification is determined by individuals who have uploaded the information to IEDB, and as such, the authors of this study did not interfere with it. However, the outcome of each assay was simply categorized as Positive (P) or Negative (N) in the majority of cases, without specifying the intensity of the response. We assimilated the ‘Positive’ value to the ‘Intermediate Positive’ value in order to use as much information as possible; and we weighted the result of the assays as: 3 (HP), 2 (IP or P), 1 (LP), and 0 (N), so that, if we call $n_{e,T,HP} , n_{e,T,IP} , n_{e,T,LP}$ and $n_{e,T,N}$ to the number of High Positive, Intermediate Positive, Low Positive and Negative T Cell Assays, respectively, for the epitope *e*, and, analogously, $n_{e,B,HP} , n_{e,B,IP} , n_{e,B,LP}$ and $n_{e,B,N}$ for B Cell Assays and $n_{e,MHC,HP} , n_{e,MHC,IP} , n_{e,MHC,LP}$ and $n_{e,MHC,N}$ for MHC Ligand Assays, we obtain the following three numbers $T_{e} , B_{e} , MHC_{e}$, which are in the interval [0*,* 1]:

$$T_{e} = \frac{{3n_{e,T,HP} + 2n_{e,T,IP} + n_{e,T,LP} }}{{3\left( {n_{e,T,HP} + n_{e,T,IP} + n_{e,T,LP} + n_{e,T,N} } \right)}}$$

$$B_{e} = \frac{{3n_{e,B,HP} + 2n_{e,B,IP} + n_{e,B,LP} }}{{3\left( {n_{e,B,HP} + n_{e,B,IP} + n_{e,B,LP} + n_{e,B,N} } \right)}}$$

$$MHC_{e} = \frac{{3n_{e,MHC,HP} + 2n_{e,MHC,IP} + n_{e,MHC,LP} }}{{3\left( {n_{e,MHC,HP} + n_{e,MHC,IP} + n_{e,MHC,LP} + n_{e,MHC,N} } \right)}}$$

A weighted mean of the numbers $T_{e} , B_{e} , MHC_{e}$ for which there are available assays (with a number of assays $n_{e,T} = n_{e,T,HP} + n_{e,T,IP} + n_{e,T,LP} + n_{e,T,N}$*,*$n_{e,B} = n_{e,B,HP} + n_{e,B,IP} + n_{e,B,LP} + n_{e,B,N}$ and $n_{e,MHC} = n_{e,MHC,HP} + n_{e,MHC,IP} + n_{e,MHC,LP} + n_{e,MHC,N}$, respectively) is then performed. The weighting is taken as the mean of these two weightings: 1) The weights of all the groups is the same, 2) The weights of the groups are proportional to the number of assays in the group. We thus consider three cases:

- 1. There is only one type G1 of assays. In this case, we take ${\text{w}}^{\prime } \left( {\text{e}} \right){ } = {\text{ G}}1_{{\text{e}}}$.
- 2. There are two types G1 and G2 of assays with a number of assays of $n_{e,G1}$ and $n_{e,G2}$, respectively. The weights are $\frac{{3n_{e,G1} + n_{e,G2} }}{{4\left( {n_{e,G1} + n_{e,G2} } \right)}}G_{1,e}$ and $\frac{{n_{e,G1} + 3n_{e,G2} }}{{4\left( {n_{e,G1} + n_{e,G2} } \right)}}G_{2,e}$, so that we take
  $$w^{\prime } \left( e \right) = \frac{{3n_{e,G1} + n_{e,G2} }}{{4\left( {n_{e,G1} + n_{e,G2} } \right)}}G_{1,e} + \frac{{n_{e,G1} + 3n_{e,G2} }}{{4\left( {n_{e,G1} + n_{e,G2} } \right)}}G_{2,e}$$
- There are three types *T, B, M HC* with a number of assays $n_{e,T} ,n_{e,B} , n_{e,MHC}$ , respectively. The weights are $\frac{{4n_{e,T} + n_{e,B} + n_{e,MHC} }}{{6\left( {n_{e,T} + n_{e,B} + n_{e,MHC} } \right)}}, \frac{{n_{e,T} + 4n_{e,B} + n_{e,MHC} }}{{6\left( {n_{e,T} + n_{e,B} + n_{e,MHC} } \right)}}$ and $\frac{{n_{e,T} + n_{e,B} + 4n_{e,MHC} }}{{6\left( {n_{e,T} + n_{e,B} + n_{e,MHC} } \right)}}$, so that we take
  $$w^{\prime } \left( e \right) = \frac{{4n_{e,T} + n_{e,B} + n_{e,MHC} }}{{6\left( {n_{e,T} + n_{e,B} + n_{e,MHC} } \right)}}T_{e} + \frac{{n_{e,T} + 4n_{e,B} + n_{e,MHC} }}{{6\left( {n_{e,T} + n_{e,B} + n_{e,MHC} } \right)}}B_{e} + \frac{{n_{e,T} + n_{e,B} + 4n_{e,MHC} }}{{6\left( {n_{e,T} + n_{e,B} + n_{e,MHC} } \right)}}MHC_{e}$$

We gave an extra value to the weight $w\left( e \right)$ of the epitope *e* when there were positive assays for both T Cell Assays and B Cell Assays, that is, when, $T_{e} > 0$ and $B_{e} > 0$. In this case, we took $w\left( e \right) = min\left\{ {1.2 w^{\prime } \left( e \right), 1} \right\}$. In another case, $w\left( e \right) = w^{\prime } \left( e \right)$.

Finally, we weighted by 0 the epitopes without positive assays for both T Cell and B cell, that is, the ones with $T_{e} = 0$ and $B_{e} = 0$, and hence we did not include those epitopes in the search performed using the algorithm.

We next used the integer programming algorithm to obtain the set of possible solutions (See Methods for more information). Table 1 depicts some of those solutions (those with lengths up to 50 amino acids); the rest of the vaccine candidates with lengths between 50 and 100 amino acids can be found in Table S1.

**Table 1:** Solutions for the optimization procedure.

*λ*	Length	String
0*.*5556	14	NGSWHYRLWHYPCT
0*.*6667	19	YPYRLWHYRMYVGGNGSWH
1*.*2223	29	YPYRLWHYPCTGLIHLHQLINTNGSWHIN
1*.*3334	32	RMYVGGNTNGSWHINDYPYRLWHYTGLIHLHQ
1*.*4949	36	PALSTGLIHLHQLINTNGSWHINDYPYRLWHYPCTV
1*.*8061	37	LPALSTGLIHLHQLINTNGSWHINDYPYRLWHYPCTV
1*.*889	39	STGLIHLHQLVNTNGSWHINRMYVGGDYPYRLWHYPCTI
1*.*8894	41	LPALSTGLIHPPLGNWFGDYPYRLWHYPCTVNTNGSWHINR
2*.*0001	42	NTNGSWHINRDYPYRLWHYALSTGLIHLHQNIVDVQYLYGVG
2*.*0815	44	YPYRLWHYLPALSTGLIHLHQNIVDVQYLYGVGNTNGSWHINRT
2*.*2061	45	LPALSTGLIHLHQLINTNGSWHINPTDCFRKHDYPYRLWHYPCTV
2*.*6668	47	LPALSTGLIHLHQNIVDVQYLYGVGDYPYRLWHYQLINTNGSWHINR

Given the considerations about the lengths stated at the beginning of the paper, we then chose the vaccine candidate with *λ* = 1*.*889 and length = 39, which is.

STGLIHLHQLVNTNGSWHINRMYVGGDYPYRLWHYPCTI

This candidate was composed of the union of 13 epitopes, more precisely, the vaccine candidate covered the following epitopes:

E1: STGLIHLHQ, E2: YRLWHYPCTI, E3: YRLWHYPCT, E4: DYPYRLWHY, E5: RLWHYPCTI, E6: NGSWH, E7: NTNGSWHIN, E8: NTNGSWHINR, E9: QLVNTNGSWHIN, E10: RMYVGG, E11: STGLIHLH, E12: TGLIHLHQ, E13: YPYRLWHY.

Table 2 shows the distribution of assays for those 13 epitopes.

**Table 2:** Distribution of assays.

Epitope	T cell				B cell				MHC			
	HP	IP	LP	N	HP	IP	LP	N	HP	IP	LP	N
STGLIHLHQ	0	1	0	1	0	0	0	0	0	0	0	0
YRLWHYPCTI	0	4	0	1	0	0	0	0	0	0	0	0
YRLWHYPCT	0	0	0	0	0	2	1	0	0	0	0	0
DYPYRLWHY	1	10	0	2	0	0	0	0	0	0	0	0
RLWHYPCTI	0	8	3	0	0	0	0	0	1	2	0	0
NGSWH	0	0	0	0	0	3	0	0	0	0	0	0
NTNGSWHIN	0	0	0	0	0	2	0	0	0	0	0	0
NTNGSWHINR	0	0	0	0	0	1	0	0	0	0	0	0
QLVNTNGSWHIN	0	0	0	0	0	3	0	0	0	0	0	0
RMYVGG	0	0	0	0	0	2	0	0	0	0	0	0
STGLIHLH	0	0	0	0	0	1	0	0	0	0	0	0
TGLIHLHQ	0	0	0	0	0	1	0	0	0	0	0	0
YPYRLWHY	0	1	0	1	2	7	0	0	0	1	0	0

Where HP indicates High- Positive, IP Intermediate-Positive, LP Low-Positive (LP), and N Negative, in the corresponding assay.

As observed, positive results were obtained for all three types of assays: specifically, five epitopes exhibited positive reactions in T cell assays, and nine epitopes yielded positive results in MHC assays. Additionally, some epitopes displayed positive reactions in multiple assay types: two epitopes were positive in both T cell and B cell assays, two were positive in T cell and MHC assays, one was positive in B cell and MHC assays, and finally, one epitope exhibited positive reactions in all three types of assays.

Since the candidate vaccine was obtained by disjunctively joining two strings, S1 (STGLIHLHQLVNTNGSWHINRMYVGG, which is the overlapping sum of epitopes E1, E9, E8, and E10 and referred to as the STG peptide in this study) and S2 (DYPYRLWHYPCTI, which is the overlapping sum of epitopes E4 and E2 and referred to as the DYP peptide), we separately synthesized both peptides, as well as E1 and E4 peptides, at a purity of 98.5% and employed them for the immunological assays.

A control was always included in all the immunological assays with mouse dendritic cells not loaded with any peptide, referred to here as DC, in order to avoid any sequence mimicry of HCV viral sequence with any putative mouse peptide sequence. Any immune response observed with DC controls over basal levels would be considered as putative molecular mimicry of HCV viral with mouse sequences.

## Experimental results in mice (proof of concept of the vaccine candidates)

Safety and immunogenicity are the two primary parameters to be evaluated in vaccine design. The safety of a vaccine platform can be explored in vitro by examining the toxicity and apoptosis of cells or in vivo by evaluating the levels of acute cytokines such as IL-1^27^ in the mice sera. HCV-synthesized peptides (DYP, E4, E1, or STG) or two non-related bacterial Listeria monocytogenes peptides with intermediate (GAPDH1-15) or no vaccine effectiveness (GAPDH1-10, here called PCONT) were loaded into macrophages (MØ) or dendritic cells (DC) to prepare DC vaccines. Safe peptides are those that provide cell viability values higher than 95–96% and induce early apoptosis at percentages lower than 5%. Moreover, safe peptides induce only basal levels of IL-1, lower than 1.5 pg/mL in the mice sera. Table 3 shows that all peptides exhibited values of cell viability between 97 and 98.5% and apoptotic percentages lower than 4.5% in both MØ and DC vaccines, with IL-1 levels ranging between 1 and 1.3 pg/mL in the mice sera inoculated either with peptides or DC vaccines. In summary, DC vaccines loaded with HCV peptides demonstrated excellent safety ranges both in vitro and in vivo.

**Table 3:** Safety of HCV peptides in vitro in macrophages and DC and in vivo*.*

Antigen	^c^Cell viability (%)	^d^Early apoptosis (%)	^e^In vivo toxicity
^a^Nt	99 ± 0.3	3 ± 0.3	1.0 ± 0.1
DYP	98 ± 0.2	4 ± 0.2	1.2 ± 0.1
E4	99 ± 0.2	3 ± 0.2	1.1 ± 0.1
STG	98.5 ± 0.1	3.5 ± 0.1	1.1 ± 0.1
E1	99 ± 0.1	3 ± 0.2	1.0 ± 0.1
PCONT	98.7 ± 0.1	3.3 ± 0.1	1.3 ± 0.2
^b^DC-DYP	97 ± 0.5	4.5 ± 0.4	1.2 ± 0.1
DC-E1	99 ± 0.3	3.5 ± 0.1	1.1 ± 0.1
DC-STG	98 ± 0.4	4 ± 0.3	1.1 ± 0.1
DC-E1	99 ± 0.2	3.5 ± 0.2	1.0 ± 0.1
DC-PCONT	97 ± 0.4	4.5 ± 0.4	1.0 ± 0.2
DC	99 ± 0.2	3 ± 0.2	1.1 ± 0.1

^a^Bone-marrow derived macrophages (MØ) were incubated with 50 µg/mL of peptides for 16 h. ^b^Bone-marrow derived dendritic cells (DC) were incubated with peptides as described in a. ^c^Cell viability was evaluated using Trypan blue staining through microscope counting of viable (non-stained) and non-viable (blue-stained) cells. Results are presented as percentages of viable cells relative to the total cell count (viable and non-viable cells) (P < 0.5). ^d^Early apoptosis was detected by staining DCs with the DNA marker 7-AAD-PE and the apoptotic marker Annexin-V-APC. Results indicate the percentages of apoptotic cells with standard deviation of triplicates (P < 0.5). ^e^In vivo toxicity assessment was conducted after intraperitoneal inoculation of mice with peptides or DCs loaded with peptides for 24 h. Results display the IL-1 levels in mouse sera, expressed in pg/mL with standard deviation of triplicates (P ≤ 0.1).

All experiments were conducted at least three times.

Immunogenicity was subsequently assessed using the following protocol to measure local immune responses through the induction of delayed type hypersensitivity (DTH) reactions. This was achieved by administering DC vaccines loaded with various peptides (see Fig. 1, panel B) via intraperitoneal (*i.p*.) inoculation for 7 days with DC-DYP, DC-E4, DC-E1, or DC-STG vaccines (10^6^ cells/mice), or peptide vaccines DYP, E4, E1, or STG (1 µg/mL). A subsequent priming was performed by inoculating the peptides into the left hind footpads of mice, with the right hind footpad serving as a basal control. Two days later, the DTH response was quantified as the difference in swelling between the left and right hind footpads.

Negative controls encompassed empty DC and DC loaded with PCONT, while positive controls consisted of DC vaccines loaded with a viral SARS-CoV2 peptide (CoVPSA) or a bacterial Listeria peptide (GAPDH1-22), both known for their effective vaccine performance^27,28^. Analysis of DTH responses (Fig. 1, panel C) revealed that DCs loaded with HCV peptides, specifically STG (DC-STG bars) or DYP (DC-DYP bars), exhibited 5.6–12-fold greater local immune responses compared to those elicited by empty DC (DC bars) or DC loaded with the bacterial control peptide (DC-PCONT bars). Those HCV peptide-loaded DCs demonstrated responses within the same 6.6–ninefold range as DCs loaded with bacterial or viral peptides known for their high vaccine effectiveness (DC-GAPDH1-22 or DC-CoVPSA bars).

It is noteworthy that DCs loaded with short peptides within the STG or DYP sequences, such as DC-E1 or DC-E4, elicited modest yet significant 1.5–1.8-fold DTH responses. This implies that cellular responses required a minimal length of 15 amino acids, as previously reported^27^. In contrast, peptide vaccines displayed negligible capacity to induce cellular responses (STG, DYP, E1, or E4 bars).

**Figure 2:**
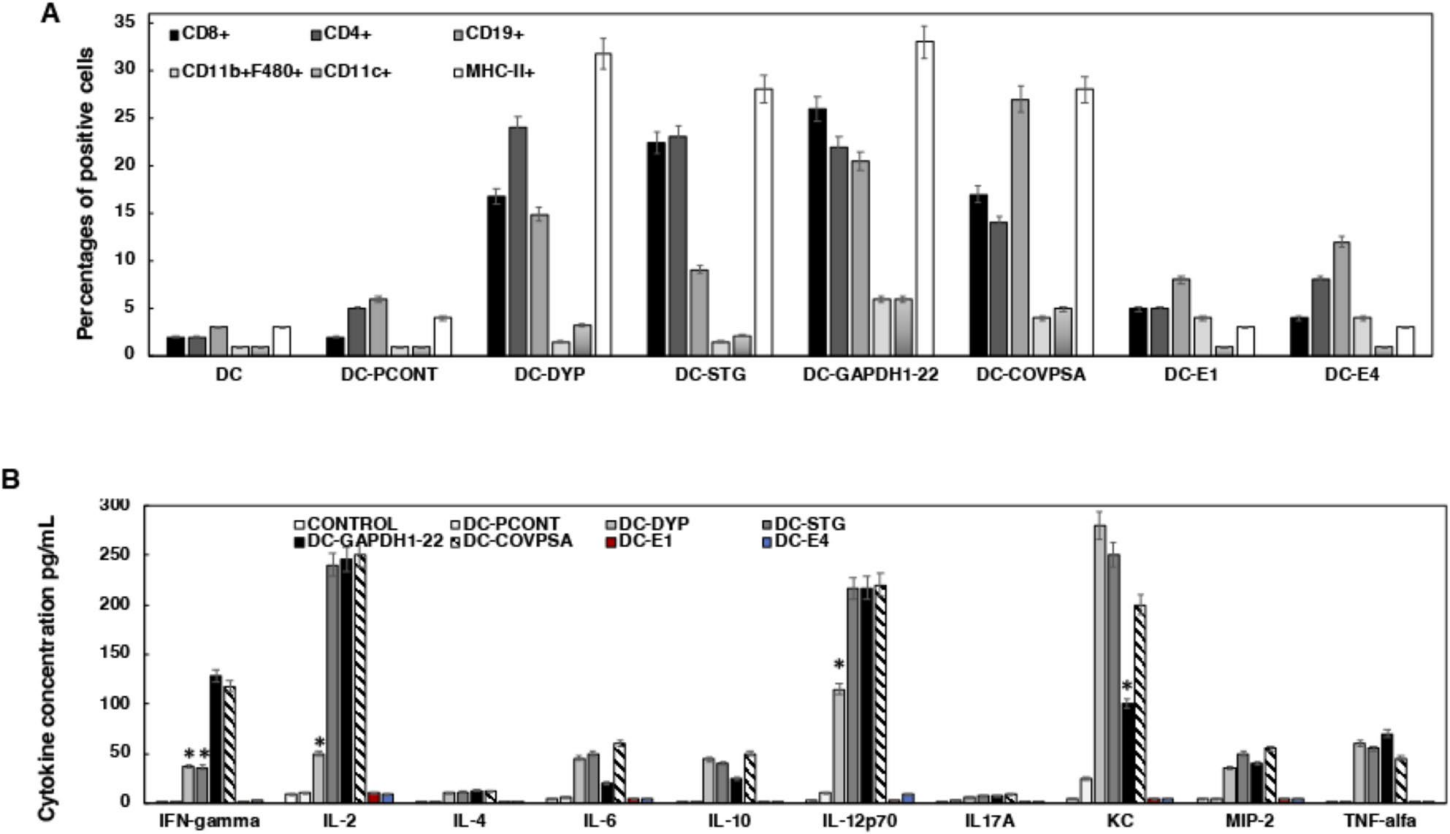
Local and systemic immune responses induced by hepatitis C peptides in DC and peptide vaccines. (**A**) Popliteal lymph nodes were isolated from the legs of mice and after homogenization, immune cell populations were analyzed by flow cytometry. The percentages of CD4 + , CD8 + T cells, CD19 + (B cells), and MHC-II positive cells, primarily representing DC or MØ, are shown. Results are expressed as the percentage of positive cells ± SD from three different experiments (P < 0.05). The experiments were performed five times. (**B**) Cytokine levels in mice inoculated with hepatitis C vaccine platforms, DC or peptide vaccines. Cytokine levels were detected in the mice sera, as described in A, and measured using a multiparametric Luminex Kit. Results are expressed as pg/mL of each cytokine ± SD of triplicate samples (P < 0.05). Cytokine experiments were conducted four times.

The immune populations within the popliteal lymph nodes were examined using flow cytometry (see Fig. 2A) to gain a more comprehensive understanding of the local immune responses triggered by the DC vaccines loaded with HCV peptides and peptide vaccines. DC vaccines loaded with HCV peptides, specifically STG or DYP, exhibited the highest proportions of immune cells characterized as MHC-II + cells (26–33%), a label typically associated with DC and MØ (macrophages). Following this, CD4 + T cells accounted for 24–25% of the immune cell population, and CD8 + T cells represented 18–22%, while CD19 + cells, which are indicative of B cells, demonstrated comparatively lower percentages ranging from 7–14%. DC vaccines loaded with HCV short peptides, such as DC-E1 and DC-E4, resulted in lower levels of T cells or DC, along with minor percentages of CD19 + cells (7–12%). In the case of DC-PCONT, the levels of CD19 + cells were 5%, and CD4 + T cells amounted to 4%. Notably, empty DC vaccines failed to induce any significant immune cell populations.

These findings suggest a strong induction of innate immune cells such as DC or MØ by DC vaccines loaded with both STG and DYP peptides, as well as specific cellular immune responses, as evidenced by the substantial presence of CD4 + and CD8 + T cells. Conversely, immune cells associated with antibody production, such as B cells, were observed in much lower quantities. The outcomes are consistent with the fact that CD4 + and CD8 + T epitopes are the primary focus of HCV vaccines. However, DC vaccines loaded with HCV short peptides DC-E1 and DC-E4 did not demonstrate the capacity to stimulate innate immune cells or T cells, but rather induced notable numbers of B cells, considering that E4 epitopes have been reported as common antibody epitopes in various studies (see Table 2).

Further confirmation of our concept was evidenced by the production of cytokines that assess the systemic immune responses stimulated by DC vaccines loaded with hepatitis C peptides in the mice sera (depicted in Fig. 2B). Notably, DC-STG vaccines elicited elevated levels of various anti-viral pro-inflammatory cytokines such as IL-2, IL-12p70, and KC. On the other hand, DC-DYP vaccines only managed to induce substantial KC levels. Effective vaccines such as DC-GAPDH1-22 or DC-CoVPSA also led to heightened IFN-gamma production. In contrast, control vaccines, namely DC-PCONT or DC, did not prompt significant levels of pro-inflammatory or anti-inflammatory cytokines. Moreover, DC-E1 and DC-E4 vaccines were incapable of inducing noteworthy pro-inflammatory cytokine levels, implying a lack of ability to induce either innate immunity or anti-viral immunity.

Lastly, the effectiveness of a vaccine is commonly associated with the generation of specific immune responses, either T cell responses, both CD4 + and CD8 + peptide-specific, or antibodies against the vaccine’s antigen. In this context, we first evaluated the frequencies of HCV peptide-specific CD4 + and CD8 + producing IFN-γ, as a measure of specific immunogenicity associated with protective DC vaccines^27,28^. DC vaccines loaded with HCV peptides that showed the highest T cell percentages, DC-DYP and DC-STG (see Fig. 2A) were selected to measure CD4 + and CD8 + producing intracellular IFN-γ in lymph nodes after in vitro peptide stimulation (5 µg/mL) for 5 h (Table 4) as described^29^. To evaluate the specificity of the assay, we included different peptides of viral or bacterial origin, the HCV peptides STG and DYP, the SARS-CoV2 peptide, CoVPSA and the bacterial GAPDH1-22 and PCONT peptides belonging to *Listeria monocytogenes*. We detected a significant 0.59% frequency of CD8 + T cells producing IFN-γ and specific for DYP peptide, but lower than the 0.8% frequency of CD8 + T cells producing IFN-γ and specific for STG peptide. The results indicate that STG peptide induces a higher and specific CD8 + T cell response than DYP peptide. Similarly, we observed a good 0.89% frequency of CD4 + T cells producing IFN-γ and specific for DYP peptide. However, we detected a higher 1.2% frequency of CD4 + T cells producing IFN-γ and specific for STG peptide (Table 4). Higher numbers of CD4 + and CD8 + epitopes in STG than in DYP peptide might explain greater CD4 + and CD8 + T cell responses, but also suggests a better DC-STG vaccine efficiency. No other viral or bacterial peptides were able to induce specific CD4 + or CD8 + T cell responses.

**Table 4:** Frequencies of peptide-specific CD4 + and CD8 + T cells producing IFN-γ in DC vaccinated mice.

Peptide/Vaccine*	GAPDH1-22	STG	DYP	CoVPSA	PCONT
DC/CD4 +	0.0% ± 0.01	0.0% ± 0.01	0.0% ± 0.01	0.0% ± 0.01	0.0% ± 0.01
DC/CD8 +	0.0% ± 0.05	0.0% ± 0.01	0.0% ± 0.01	0.0% ± 0.01	0.0% ± 0.01
DC-STG/CD4 +	0.0% ± 0.02	1.2% ± 0.02	0.1% ± 0.1	0.0% ± 0.01	0.0% ± 0.01
DC-STG/CD8 +	0.0% ± 0.01	0.8% ± 0.01	0.0% ± 0.01	0.0% ± 0.02	0.0% ± 0.11
DC-DYP/CD4 +	0.0% ± 0.01	0.0% ± 0.01	0.89% ± 0.02	0.0% ± 0.01	0.0% ± 0.01
DC-DYP/CD8 +	0.0% ± 0.01	0.0% ± 0.01	0.59% ± 0.01	0.0% ± 0.01	0.0% ± 0.01

*Mice were vaccinated (*i.p*) with DC vaccines loaded with long HCV peptides (STG or DYP) or empty DC (DC) were challenged in the footpads (f.p) with each peptide in the presence of DIO-1 adjuvant. Collected lymph nodes were cultured (5 × 10^6^ cells/mL) and stimulated with different peptides, GAPDH1-22, CoV-PSA, DYP or STG (5 µg/mL each peptide) for 5 h^29^ (see Methods). Cells were surface labelled for CD4, or CD8, fixed and permeabilized before staining for intracellular IFN-γ. FACS analysis shows the percentages of cells expressing IFN-γ and corrected to the percentages of total CD4 + or CD8 + cells. Samples were tested in triplicate and the results are the mean ± SD of two separate experiments.

Next, we assessed the elicitation of specific antibodies by DC vaccines loaded with hepatitis C peptides (summarized in Table 5).

**Table 5:** Induction of specific and cross-reactive antibodies by DC vaccines loaded with HCV peptides.

Peptide/Vaccine	GAPDH1-22	STG	DYP	E1	E4	GAPDH1-15	CoVPSA	PCONT
DC	0.248 ± 0.1	0.232 ± 0.1	0.275 ± 0.1	0.211 ± 0.1	0.189 ± 0.1	0.153 ± 0.1	0.216 ± 0.1	0.153 ± 0.1
*DC-GAPDH_1-22_	1.262 ± 0.2	0.284 ± 0.1	0.297 ± 0.1	0.201 ± 0.1	0.201 ± 0.1	0.815 ± 0.1	0.427 ± 0.1	0.515 ± 0.1
*DC-STG	0.301 ± 0.2	1.491 ± 0.2	0.536 ± 0.1	0.802 ± 0.1	0.231 ± 0.1	0.117 ± 0.1	0.239 ± 0.1	0.117 ± 0.1
DC-DYP	0.341 ± 0.1	0.281 ± 0.1	1.221 ± 0.2	0.221 ± 0.1	0.899 ± 0.1	0.169 ± 0.1	0.285 ± 0.1	0.269 ± 0.1
DC-E1	0.221 ± 0.1	0.875 ± 0.1	0.201 ± 0.1	1.381 ± 0.1	0.221 ± 0.1	0.220 ± 0.1	0.210 ± 0.1	0.198 ± 0.1
DC-E4	0.198 ± 0.1	0.245 ± 0.1	0.899 ± 0.1	0.211 ± 0.1	1.399 ± 0.1	0.179 ± 0.1	0.113 ± 0.1	0.112 ± 0.1
DC-PCONT	0.267 ± 0.1	0.234 ± 0.1	0.271 ± 0.1	0.199 ± 0.1	0.222 ± 0.1	0.613 ± 0.1	0.315 ± 0.1	0.513 ± 0.2
DC-CoVPSA	0.282 ± 0.1	0.348 ± 0.2	0.291 ± 0.1	0.256 ± 0.1	0.195 ± 0.1	0.196 ± 0.1	1.088 ± 0.2	0.196 ± 0.1

ELISA peptide expressed as OD450 ≥ 0.180 ± 0.01 (anti-GAPDH1-22, anti-GAPDH1-15, anti-STG, anti-DYP, anti-E1, anti-E4, anti-CoVPSA or anti-PCONT.

Our findings revealed that DC vaccines loaded with the STG peptide showed elevated levels of IgG antibodies against STG, along with significant levels of anti-DYP antibodies. On the other hand, DC vaccines loaded exclusively with the DYP peptide induced substantial IgG anti-DYP antibody levels, but no significant levels of anti-STG antibodies were observed. This pattern was replicated by DC vaccines loaded with GAPDH1-22, which elicited elevated levels of anti-GAPDH1-22 antibodies, along with significant levels of anti-GAPDH1-15 antibodies and anti-GAPDH1-10 (PCONT) antibodies, indicating potential for cross-reactivity. In contrast, DC vaccines loaded with the CoVPSA peptide solely generated specific IgG antibodies against CoVPSA, without any observed cross-reactions. Furthermore, DC vaccines loaded with the DC-E1 peptide – a short sequence within STG – led to specific IgG antibodies against both STG and E1. Similarly, DC-E4 vaccines loaded with a short sequence within DYP induced specific IgG antibodies against both DYP and E4, underscoring their high specificity. The results also indicate that DC-STG vaccine was specific as they only induce significant immune responses against shorter peptides such as DYP or E1 containing peptide sequences of STG but not against E4 that was not an epitope included in STG sequence. It should note that DC-STG vaccines did not induce immune responses against other peptides, bacterial (GAPDH1-15, GAPDH1-22) or viral peptides (COVPSA), which is further proof of their specificity.

In summary, our research led to the conclusion that DC vaccines loaded with STG peptides exhibited greater efficacy compared to those loaded with DYP. This conclusion is supported by several factors. First, the former induced significantly higher levels of anti-viral cytokines, signifying a robust immune response. Second, they generated elevated levels of STG-specific CD4 + and CD8 + T cell responses as well as IgG antibodies that recognized not only the primary antigen (STG) but also a portion of the antigen (E1 and DYP). This indicates their potential for cross-reactions and their capacity to function as a universal vaccine against HCV, while preserving high specificity for T cell responses. This observation is consistent with bacterial vaccines, such as DC-GAPDH1-22, which demonstrated both cross-reactive and specific capabilities, as evidenced by the high levels of IgG antibodies recognizing parts of the GAPDH1-15 or GAPDH1-10 antigens, but also highly specific T cell responses^27–29^^.^

## Discussion

This paper presents a procedure to design a vaccine capable of offering protection against all virus variants considered. By requiring the λ-superstring criterion on the optimization algorithm, we were able to design candidates that maximize the "best" covered epitopes for each Hepatitis C virus E2 protein version.

We weighted the epitopes using formulas based on the success ratio of immunological assays in order to approach the biological reality as closely as possible. The equations aim to emphasize the epitopes with the best results, giving extra value to those with positive T-cell and B-cell assays simultaneously.

However, although we targeted HCV, the methodology presented here can be used against other viruses (preferably those with high variability), such as influenza^15^, HIV^16^, or SARS CoV-2^30^.

The limitations of our technique include the computational cost of evaluating many variants and epitopes. The numbers were relatively small (1803 variants and 439 epitopes) for this paper, but if we considered all the variants of a protein, such as the S protein of the SARS CoV-2, we could have more than a million host strings, which would require supercomputing centers or similar to obtain results. One possible solution to this problem could be using a heuristic algorithm^31^ instead of the IP applied here to obtain approximate solutions close enough to the best one.

We here present evidence that DC vaccines loaded with two HCV peptides, STG (a longer peptide) and DYP (a shorter peptide), are safe and immunogenic vaccines. We validated DC vaccines loaded with the STG peptide as more efficient vaccines than those loaded with the DYP peptide, as they induce higher ranges of T cell immune responses (both CD4 + and CD8 +) as well as innate immune cells. The effectiveness of the DC vaccines loaded with STG peptide was validated as they produced high levels of anti-viral cytokines such as IL-2, IL-12p70, and KC and specific IgG antibodies anti-STG but also presented abilities as cross-reactive antibodies anti-DYP, anti-E1 and anti-E4 shorter versions of STG and DYP peptide sequences, respectively, while inducing high frequencies of STG specific CD4 and CD8 T cell responses. A common capacity detected in bacterial vaccines proposed as universal vaccines as DC loaded with GAPDH1-22 peptide^27–29^. We conclude that DC vaccines loaded with STG peptide are a promising vaccine to be implemented in vaccine platforms of easy scaling up such nanoparticles with abilities for universal vaccines for hepatitis C.

It is important to emphasize that we intentionally focused throughout our study, on utilizing human HCV sequences exclusively for both peptide and DC vaccine platforms, as well as for all experimental procedures. Although we acknowledge that this approach might have potentially impacted the extent of our immunogenicity outcomes and even the vaccine's overall effectiveness, our decision was driven by the aim of establishing a vaccine candidate and platform that could be directly applicable to human immune cells derived from both control donors and individuals with HCV infections.

There are no commercially available HCV vaccines, only several trials with vaccines that have tried to elicit HCV-specific T cell responses. The platforms and approaches included DNA-based platforms using nonstructural regions of the HCV genome, virus-like particles (VLPs) with E1 or E2 proteins, E1E2 proteins, hepatitis B virus surface antigen-HCV recombinants, and pooled synthetic class I peptide epitopes or peptides incorporated in lysosomes^27^. To date, DNA vaccines have induced antibodies and some T cell responses, but they do not induce sterilizing immunity. VLP vaccines do not induce antibodies, or do class I peptide epitopes or peptides incorporated in lysosomes^32^. In this regard, DC-STG vaccines are prepared with an E2 glycoprotein combination of peptides that induce innate immune responses, cellular responses, as well as antibody responses. Therefore, they have shown a high potential to be tested in human immune cells to complete the preclinical studies, and if successful, be proposed for human trials.

In summary, our study has introduced and demonstrated the efficacy of a novel vaccine design approach that holds the potential to be extrapolated to combat other viruses characterized by a high mutation rate and the absence of a viable vaccine solution. This innovative procedure offers a promising avenue for addressing the challenges posed by such viruses and holds the promise of advancing vaccine development in these critical areas.

## Methods

### Weighted λ-superstring

In short, the definition of weighted *λ*-superstring is the following:

#### Definition 2.1.

*Let* $H, T \subseteq A^{*}$ *be two finite sets of host and target strings, respectively, let each target string* ${\varvec{t}} \in T$ *be equipped with a weight* $w\left( {\varvec{t}} \right) \in {\mathbb{R}}$*, and let* $\lambda \in {\mathbb{R}}$*. A weighted λ-superstring for* $\left( {H, T, w} \right)$ *is a string* ${\varvec{v}} \in A^{*}$ *such that for every* $\user2{h } \in H$*, the sum of the weights of the target strings that are common substrings of both* ${\varvec{h}}$ *and* ${\varvec{v}}$ *is at least λ.*

In short, a weighted *λ*-superstring ${\varvec{v}}$ is a string constructed in a way that, for every host string ${\varvec{h}}$ in set *H*, it ensures that the sum of the weights of target strings ${\varvec{t}}$ common to both ${\varvec{h}}$ and ${\varvec{v}}$ is greater than or equal to *λ*. For more information related to the properties of *λ*-superstrings, see Martinez et.al^15,16,25^.

### Sample acquisition

First, , we used the extract tool provided^34^ to search the European HCV Database^33^ to obtain all possible variants of the E2 protein of the Hepatitis C virus More precisely, our reference was the E2 protein with code “AF009606_E2_C1a”, with the following sequence:

ETHVTGGSAGRTTAGLVGLLTPGAKQNIQLINTNGSWHINSTALNCNESLNTGWLAGLFYQHKFNSSGCPERLASCRRLTDFAQGWGPISYANGSGLDERPYCWHYPPRPCGIVPAKSVCGPVYCFTPSPVVVGTTDRSGAPTYSWGANDTDVFVLNNTRPPLGNWFGCTWMNSTGFTKVCGAPPCVIGGVGNNTLLCPTDCFRKHPEATYSRCGSGPWITPRCMVDYPYRLWHYPCTINYTIFKVRMYVGGVEHRLEAACNWTRGERCDLEDRDRSELSPLLLSTTQWQVLPCSFTTLPALSTGLIHLHQNIVDVQYLYGVGSSIASWAIKWEYV VLLFLLLADARVCSCLWMMLLISQAEA.

Furthermore, we used the default values in the search of $\pm 5\%$ on the allowed variation of extracted sequence length and of 0.01 in the frequency threshold above which residues are shown.

A total of 2126 sequences appeared and, after removing repeated sequences, 1803 sequences were obtained, which formed the set of host strings in the search of a weighted *λ*-superstring.

The set of target strings was next taken to be the set of epitopes appearing in the Immune Epitope Database and Analysis Resource^35^. The search was performed using “Envelope glycoprotein E2 (384–746) [PRO 0000037570] (Hepatitis C virus)” in the “Antigen Name” field of the “Antigen” window, “Humans” in the “Host” window and “Linear Epitope” in the “Epitope” window. The reason to consider only linear epitopes is that the concept of weighted *λ*-superstrings is addressed primarily to sets of host strings and target strings which are linear sequences in an alphabet; hence we are interested in the primary structure of the corresponding proteins (host strings) and peptides (target strings). Given that cellular and humoral responses are generated during acute infection with HCV^10^, we considered epitopes for which T-cell assays have been performed and likewise epitopes with B-cell assays. We obtained 439 epitopes in the search. Two of the epitopes, QLINTNGSWHVN and QLINTNGSWHVN + Pyre Q1, with epitope IDs 51,374 and 176,383, respectively, in IEDB, are based on the same peptide. The second one is a complex of AP33 Fab with the peptide^36^; hence we did not include it in the set of target strings since in the framework of *λ*-superstrings we only considered linear sequences of amino acids (aa).

### Optimization procedure

We used the integer programming algorithm described in Martinez et al.^14^ to solve the optimization problem and obtain the best possible vaccine candidate according to the weighting described above In summary, the goal is to maximize the value of λ for a fixed length, using the set of host strings described in "Sample acquisition" and the set of target strings and weights described in the Results section. The Integer Programming problem proposed to obtain the best vaccine candidates is based on a graph-theoretic formulation of the optimization problem, which is a generalization of the Traveling Salesman Problem^14^. More specifically, we used the algorithm to maximize, for a given value of *n*, the value of *λ* for *λ*-superstrings no longer than 100. In our case, *λ* represents the value of the minimum sum of the weights included both in the vaccine candidate and in each virus variant. Therefore, we obtain strings that “protect” at least “*λ”* against every virus variant.

The Integer Programming algorithm described above was solved using CPLEX Optimizer 39, running on an Intel(R) Xeon(R) CPU E5-4620 v2 @ 2.60 GHz Processor with 512 GB of RAM.

### Peptides, adjuvants and mice

The sequence of the two peptides from the E2 protein, STG that comprises the overlapping epitopes of E1, E8, E9 and E10 with the following sequence STGLIHLHQLVNTNGSWHINRMYVGG, and the peptide DYP that comprises the overlapping epitopes of E3 and E4 with the following sequence, DYPYRLWHYPCTI, the peptides E1 and E4 as well as two other non-related bacterial peptides of glyceraldehyde-3-phosphate-dehydrogenase, a peptide with vaccine effectiveness, GAPDH1-22 with sequence, MTVKVGINGFGRIGRLAFRRIQ and a peptide with no vaccine effectiveness, GAPDH1-10 here called PCONT, MTVKVGINGF, were all synthesized by Genescript with a purity ≥ 99% as estimated by HPLC. DIO-1 adjuvant was a gift of M. Fresno (CBMSO, Madrid) and used at a concentration of 2 µg/mL.

Mice were 8–12-week-old female C57BL/6 from Charles River (France).

### Preparation of immune cells to evaluate safety of HCV peptides

Bone-marrow derived dendritic cells (DC) or macrophages (MØ were obtained from femurs of 8–12 female mice and cultured at 2 × 106 cells/mL in six-well plates in Dulbecco´s Modified Eagle´s Medium (DMEM) supplemented with 20% fetal calf serum, 1 mM glutamine, 1 mM nonessential amino acids, 50 µg/mL gentamicin, 30 µg/mL vancomycin (DMEM complete medium) and 20 ng/mL of granulocyte-colony-stimulating factor (GM-CSF) to obtain DC or with 20 ng/mL of macrophage-colony-stimulating factor (M-CS) to obtain MØ. On day 7, cells were collected and examined by flow cytometry to evaluate cell surface markers. Differentiated DC showed a phenotype of 98% CD11c^+^MHC-II^+^CD11b^-/+^CD40^-^CD86^-^, while differentiated BM-DM showed a phenotype of 99% CD11c^-^MHC-II^+/-^CD11b^+^F4/80^-^. Those cells were used to evaluate the safety of HCV peptides.

Safety of cells loaded with HCV peptides (MØ or DC) was explored with two assays in vitro, cell viability and apoptosis and the in vivo assay measuring the levels of IL-1 in mice sera. Safety was considered optimal if the percentages of cell viability were higher than 95% and apoptosis induction was no higher than 5%. Cell viability was explored after BM-DM or DC incubation with synthesized peptides at a concentration of 50 µg/mL for 16 h, washed, and stained with Trypan blue. Results were expressed as the percentage viability compared with non-treated BM-DM or DC of triplicate experiments (P < 0.05). Apoptosis was measured by labelling the DNA with a fluorescent intercalating probe, 7-aminoactinomycin D (7-ADD, BD Biosciences, San Jose, CA, USA) and analysis for the cell surface marker of apoptosis, Annexin-V conjugated to allophycocyanin (APC) fluorochrome, followed by incubating the cells for 16 h with the peptides. Staining of cells with 7-ADD corresponded to normal cell death and staining with annexin-V alone corresponds to the apoptotic cell death. The results are expressed as the mean ± SD (P < 0.05). Safety of HCV peptides in vivo was explored after intraperitoneal inoculation of 50 µg/mL of peptides alone or DC loaded peptides (2 × 10^6^ cells/mice of DC vaccines) for 24 h. Mice were bled and sera obtained and analyzed for IL-1 cytokine as a measurement of an acute cytokine after a toxic inoculation. IL-1 concentration was used using the CBA proinflammatory kit (BD Bioscience, NJ, USA) and expressed as the average of three replicates in pg/mL ± SD.

### Preparation of DC vaccines loaded with HCV peptides

DC cells prepared as in previous section were loaded ex vivo with 50 µg/mL of GAPDH1-10 (PCONT), GAPDH1-22, STG or DYP peptides for 24 h in the presence of 40 ng/mL of DIO-1. Cells were washed and used as DC vaccines for in vivo experiments.

### T cell responses elicited by DC-vaccines loaded with HCV peptides or peptide vaccines

To perform delayed type hypersensitivity (DTH) analysis, C57BL/6 mice were first primed with HCV peptides (50 µg/mL) intraperitoneally (i.p) for 7 days. We then inoculated with DC vaccines loaded with different peptides (STG, E1, DYP, E4, PCONT, or GAPDH1-22) or none (DC) into the left hind footpad (10^6^ cells/mice) or peptide vaccines inoculated directly (1 µg/mL). The right hind footpads served as negative controls since they were not inoculated. After 48 h, we measured the footpad thickness using a caliper and expressed the results in millimeters as the mean of three different experiments. Subsequently, we collected and homogenized the popliteal lymph nodes and passed cell homogenates through cell strainers to analyze CD4 + and CD8 + T cells by flow cytometry. The percentage of positive cells ± SD expresses the results.

Lymph nodes were cultured in 96-well plates (5 × 10^6^ cells/mL) and stimulated with different peptides – GAPDH1-22, CoVPSA, PCONT, DYP or STG (5 µg/mL each peptide) for 5 h in the presence of brefeldin A as reported^27,37^ – to measure CD4 + or CD8 + frequencies producing intracellular IFN-γ after DC vaccination with HCV peptides STG or DYP (f.p) Cells were then surface labelled for CD4 or CD8, fixed and permeabilized with cytofix/cytoperm kit before staining for intracellular IFN-g (BD Biosciences). FACS data were analyzed using FlowJo software (Treestar, Ashland, OR). After sample acquisition, data were gated for CD4 + or CD8 + events, and the percentages expressing IFN-g determined according to the manufacturer´s recommendations. Results were corrected according to the percentages of total CD4 + or CD8 + cells. Samples were tested in triplicate and results are the mean ± SD of two separate experiments.

### Cytokine measurements

Multiparametric Luminex kits were used to quantify cytokines in mouse sera. The levels of Interferon gamma (IFN-gamma), IL-2, IL-4, IL-6, IL-10, IL-12(p70), IL-17A, KC/CXCL1, MIP-2 and TNF-α in mice serum samples were quantified using the Luminex 200 platform with a magnetic system (Milliplex MAP Mouse High Sensitivity T Cell Magnetic Bead Panel, EMP Millipore Corporation, Billerica, MA, USA), following the manufacturer's instructions. The cytokine concentrations were expressed as the average of three replicates in pg/mL ± SD.

### ELISA measurements of antibodies

Antibodies of IgG isotype against the HCV peptides, STG and DYP, negative control bacterial peptide PCONT (GAPDH1-10), bacterial positive control peptide (GAPDH1-22) and COVID positive control peptide (COVPSA) were assessed according to previously reported methods^21,23^. In brief, ninety-six well plates were coated with the different peptides at a concentration of 50 µg/mL in carbonate buffer (pH 8.0) at 4ºC overnight. Plates were then washed and incubated with 1 mg/mL of BSA (fraction V) for blocking non-specific sites. The mice sera inoculated with DC vaccines loaded with the different peptides and controls were 1/10 diluted and peptide-coated plates were incubated with the diluted sera for two hours at room temperature. Reactions were developed with goat anti-mouse IgG and the absorbance analyzed at 450 nm and expressed as optical units (OD) from the mean values ± SD of triplicate experiments (P < 0.05). Only results with OD ≥ 0.2 ± 0.1 were considered positive.

### Statistical analysis

As the number of measurements was less than 30, we first studied the normality of the value distributions using the Shapiro–Wilk test. Since all samples were normally distributed, Student´s t test was applied to all mouse assays and ELISA assays. All samples were evaluated in triplicate and experiments were performed at least three times. GraphPad software was used for generation of all graphs presented.

### Ethics statement

This study was carried out in accordance with the Guide for the Care and Use of Laboratory Animals of the Spanish Ministry of Science, Research and Innovation. Furthermore, it was performed in accordance with ARRIVE guidelines (https://arriveguidelines.org). The Committee on the Ethics of Animal Experiments of the University of Cantabria approved the protocol (Permit number: PI-10–17) pursuant to Spanish legislation (RD 1201/2005). All surgeries were performed by cervical dislocation¡ and all efforts were made to minimize animal suffering.

### Supplementary Information

Supplementary Information.

## Data Availability

All data is available in the manuscript or the Methods.
