# Supplementary material for: Testing a vaccine candidate against Hepatitis C virus designed by combinatorial optimization
Source: Sci Rep. 2023 Dec 8;13:21746. doi: 10.1038/s41598-023-48458-x (PMC10709393; doi:10.1038/s41598-023-48458-x)
Supplement: Supplementary file 1 — Supplementary Information. [file 41598_2023_48458_MOESM1_ESM.docx]

**Supplementary information**

Supplementary Table S1. Solutions for the optimization procedure with lengths 50-100aa.

| *λ* | length | string |
| --- | --- | --- |
| 2*.*6712 | 53 | QLINTNGSWHINRDYPYRLWHYLPALSTGLIHLHQNIVDVQYLYGVGSGMVGW |
| 2*.*9557 | 55 | NTNGSWHINRYPYRLWHYPCTVLPALSTGLIHLHQNIVDVQYLYGVGPTDCFRKH |
| 3*.*2268 | 56 | DYPYRLWHYPCTLPALSTGLIHLHQNIVDVQYLYGVGLTSLFTQLINTNGSWHINR |
| 3*.*3038 | 60 | QLINTNGSWHINRCLVDYPYRLWHYPCTVLPALSTGLIHLHQNIVDVQYLYGVGLTSLFT |
| 3*.*3335 | 61 | LPALSTGLIHLHQNIVDVQYLYGVGLTSLFTQLINTNGSWHINRTCLVDYPYRLWHYPCTV |
| 3*.*6268 | 64 | QLINTNGSWHINRPTDCFRKHDYPYRLWHYPCTLPALSTGLIHLHQNIVDVQYLYGVGSGMVGW |
| 3*.*7038 | 68 | CLVDYPYRLWHYPCTVQLINTNGSWHINRLPALSTGLIHLHQNIVDVQYLYGVGLTSLFTPTDCFRKH |
| 3*.*7335 | 69 | QLINTNGSWHINRTCLVDYPYRLWHYPCTVLPALSTGLIHLHQNIVDVQYLYGVGLTSLFTPTDCFRKH |
| 3*.*8518 | 70 | RMYVGGLTSLFTLPALSTGLIHLHQNIVDVQYLYGVGSGMVGWQLINTNGSWHINRTDYPYRLWHYPCTI |
| 3*.*8891 | 72 | DYPYRLWHYPCTVFNSSGCPERMYVGGQLVNTNGSWHINRTLPALSTGLIHLHQNIVDVQYLYGVGSGMVGW |
| 3*.*8935 | 74 | DYPYRLWHYPCTVGLTSLFTFNSSGCPERQLINTNGSWHINRTLPALSTGLIHLHQNIVDVQYLYGVGSGMVGW |
| 3*.*9585 | 77 | QLINTNGSWHINRTLPALSTGLIHLHQNIVDVQYLYGVGSGMVGWLTPRCLVDYPYRLWHYPCTVRMYVGGLTSLFT |
| 4*.*0002 | 79 | QLINTNGSLPALSTGLIHLHQNIVDVQYLYGVGLTSLFTCLVDYPYRLWHYPCTVNFNSSGCPERQLVNTNGSWHINRT |
| 4*.*2891 | 80 | PALSTGLIHLHQNIVDVQYLYGVGSGMVGWDYPYRLWHYPCTVPTDCFRKHKFNSSGCPERMYVGGQLVNTNGSWHINRT |
| 4*.*2935 | 82 | PTDCFRKHFNSSGCPERDYPYRLWHYPCTVGLTSLFTLPALSTGLIHLHQNIVDVQYLYGVGSGMVGWQLINTNGSWHINRT |
| 4*.*3585 | 85 | QLINTNGSWHINRTCLVDYPYRLWHYPCTVFNSSGCPERPTDCFRKHLPALSTGLIHLHQNIVDVQYLYGVGSGMVGWGLTSLFT |
| 4*.*3705 | 86 | QLINTNGSWHINRTLPALSTGLIHLHQNIVDVQYLYGVGLTSLFTCLVDYPYRLWHYPCTVNFNSSGCPERPYCWHYAPTDCFRKH |
| 4*.*4002 | 87 | QLINTNGSLPALSTGLIHLHQNIVDVQYLYGVGLTSLFTPTDCFRKHCLVDYPYRLWHYPCTVNFNSSGCPERQLVNTNGSWHINRT |
| 4*.*5185 | 89 | QLVNTNGSWHINRTFNSSGCPERMYVGGDYPYRLWHYPCTIHPEATYSRCLPALSTGLIHLHQNIVDVQYLYGVGSGMVGWPYCWHYAP |
| 4*.*5558 | 92 | QLVNTNGSWHINRTALNLTPRCLVDYPYRLWHYPCTVNFLPALSTGLIHLHQNIVDVQYLYGVGSGMVGWHKFNSSGCPERMYVGGLTSLFT |
| 4*.*6252 | 94 | LTPRCLVDYPYRLWHYPCTVNFNSSGCPERMYVGGLTSLFTQLVNTNGSWHINRTQLINTNGSLPALSTGLIHLHQNIVDVQYLYGVGSGMVGW |
| 4*.*6525 | 95 | CLVDYPYRLWHYPCTVNFPTDCFRKHKFNSSGCPERMYVGGLTSLFTQLVNTNGSWHINRTALNLPALSTGLIHLHQNIVDVQYLYGVGSGMVGW |
| 4*.*9558 | 99 | LPALSTGLIHLHQNIVDVQYLYGVGSGMVGWQLVNTNGSWHINRTALNPTDCFRKHKFNSSGCPERMYVGGLTSLFTLTPRCLVDYPYRLWHYPCTVNF |
